# Substrate Specificity of SARS-CoV-2 Nsp10-Nsp16 Methyltransferase

**DOI:** 10.3390/v13091722

**Published:** 2021-08-30

**Authors:** Roberto Benoni, Petra Krafcikova, Marek R. Baranowski, Joanna Kowalska, Evzen Boura, Hana Cahová

**Affiliations:** 1 Institute of Organic Chemistry and Biochemistry of the Czech Academy of Sciences, 16610 Prague, Czech Republic; roberto.benoni@uochb.cas.cz (R.B.); krafcikova@uochb.cas.cz (P.K.); 2 Division of Biophysics, Institute of Experimental Physics, Faculty of Physics, University of Warsaw, Ludwika Pasteura 5, 02-093 Warsaw, Poland; marek.baranowski@fuw.edu.pl (M.R.B.); Joanna.Kowalska@fuw.edu.pl (J.K.)

**Keywords:** virus, SARS-CoV-2, methylation, inhibitor

## Abstract

The ongoing COVID-19 pandemic exemplifies the general need to better understand viral infections. The positive single-strand RNA genome of its causative agent, the SARS coronavirus 2 (SARS-CoV-2), encodes all viral enzymes. In this work, we focused on one particular methyltransferase (MTase), nsp16, which, in complex with nsp10, is capable of methylating the first nucleotide of a capped RNA strand at the 2′-O position. This process is part of a viral capping system and is crucial for viral evasion of the innate immune reaction. In light of recently discovered non-canonical RNA caps, we tested various dinucleoside polyphosphate-capped RNAs as substrates for nsp10-nsp16 MTase. We developed an LC-MS-based method and discovered four types of capped RNA (m^7^Gp_3_A(G)- and Gp_3_A(G)-RNA) that are substrates of the nsp10-nsp16 MTase. Our technique is an alternative to the classical isotope labelling approach for the measurement of 2′-O-MTase activity. Further, we determined the IC_50_ value of sinefungin to illustrate the use of our approach for inhibitor screening. In the future, this approach may be an alternative technique to the radioactive labelling method for screening inhibitors of any type of 2′-O-MTase.

## 1. Introduction

The severe acute respiratory syndrome coronavirus 2 (SARS-CoV-2) is the causative agent of the current COVID-19 pandemic [1] that has already infected more than 170 million people and claimed over 3.5 million lives, according to the World Health Organization (WHO). It belongs to the *Coronaviridae* family that has already produced at least two other deadly human viruses during the last two decades. The severe acute respiratory syndrome (SARS) virus was identified as the virus causing atypical pneumonia in the Guangdong Province of China in 2002 [2], and the Middle East Respiratory Syndrome (MERS) virus was responsible for the outbreak of a respiratory disease in 2012 in the Arabian Peninsula region [3].

Coronaviruses are now recognized as a major threat to global human health [4]. Their genome is a single-stranded positive-sense RNA that encodes four structural and sixteen non-structural (nsp1-16) proteins [5]. The non-structural proteins perform most of the enzymatic activity essential for the viral life cycle that is not available in the host cells. The non-structural proteins are the RNA-dependent RNA-polymerase (RdRp); the two proteases, papain-like protease (PL^pro^) and 3C-like main proteases (3CL^pro^); the nsp13 helicase; the nsp15 endonuclease; and two methyltransferases [5]. Each of these enzymes is a potential target for antivirals [6]. Therefore, SARS-CoV-2 enzymes are intensively studied. The prime target is the RdRp, a heterotrimeric protein complex composed of nsp7, nsp8, and nsp12. The only small molecule currently approved for experimental treatment by the FDA, remdesivir, inhibits the RdRp [7], although the promising oral drug candidate PF-07321332 by Pfizer, which targets the SARS-CoV-2 protease, has just entered clinical trials. The RdRp has been structurally well characterized, including its interaction with RNA and with remdesivir [8,9,10,11]. Further, the structure and first inhibitors of the main protease 3CL^pro^ were recently described [12]. In addition, the first structures of the MTases were solved [13,14,15,16], and first inhibitors were synthesized by us and others [17,18,19].

**Figure 1:**
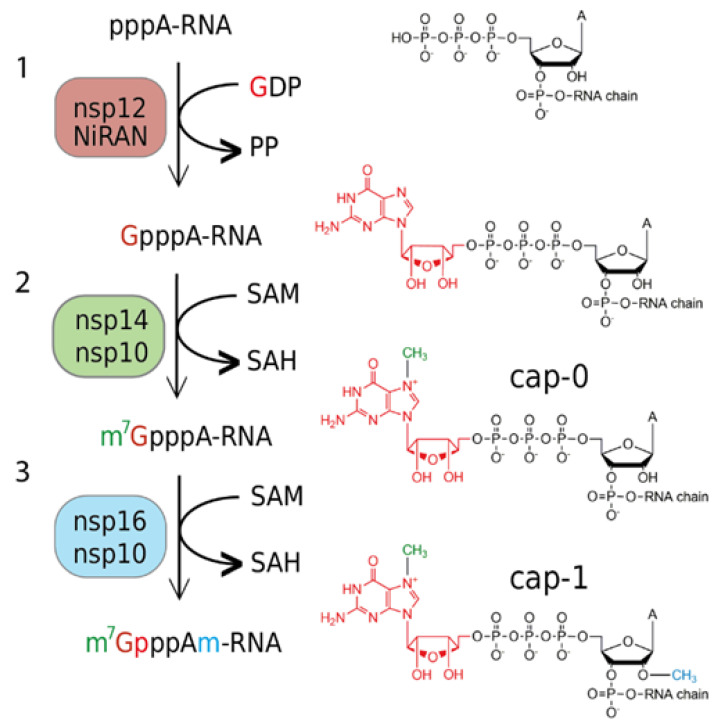
Overview of the cap 1 structure formation in SARS−CoV-2: (**1**) The NiRAN domain of nsp12 polymerase subunit catalyzes the transfer of GDP to the nascent RNA (5′pppA−RNA), releasing a pyrophosphate; (**2**) nsp14 methyltransferase with a co−factor nsp10 methylates guanosine at the N7 position and forms the cap−0 structure (m7GpppA); (**3**) nsp16 in complex with nsp10 methylates ribose at the 2′O position of the first transcribed nucleotide to form the cap−1 structure (m7GpppAm).

Innate immunity is a crucial part of the human immune system, and viruses have evolved abilities to evade it [20]. The 5′-end of the nascent RNA is a part of the pattern recognized by the RIG-I (retinoic acid-inducible gene I) pattern recognition receptor. It recognizes short viral dsRNA with a 5′-triphosphate [21] or 5′-diphosphate [22], which leads to interferon (IFN) expression. Subsequently, IFN-induced proteins with tetratricopeptide repeats 1 and 5 (IFIT 1 and IFIT5) sequester uncapped (5′-triphosphorylated) and 5′-capped RNAs lacking 2′-O-methylation at the first transcribed nucleotide (RNA carrying cap-0), which prevents binding to the eukaryotic translation initiation factor 4E (EIF4E) and inhibits its translation [23]. Coronaviruses have two RNA MTases, nsp14 and nsp16, that ensure the creation of the RNA cap (Figure 1). Nsp14 is an N7-MTase that methylates the first GTP nucleobase and, subsequently, nsp16, a 2′-O-MTase methylates the following nucleotide. Interestingly, the SARS-CoV nsp16 is only active when it is in complex with nsp10, which acts as its activation factor [24].

The chemical variations in RNA caps and their physiological implications are not fully understood. Recently, it has been shown that, beside the common canonical m^7^Gp_3_N cap, RNA can be capped by cofactors, such as nicotinamide adenine dinucleotide [25,26] or coenzyme A [27,28]. Whereas the regulatory role of the NAD-cap in bacteria has been partially elucidated [29], its function in mammalian cells is not yet fully understood [30], albeit it was suggested that it promotes RNA decay [26]. The role of the CoA-cap is unknown. Recently, we reported the discovery of an entirely new class of 5′ RNA caps in bacteria [31]. These caps have the structure of dinucleoside polyphosphates (Np*_n_*Ns) and are incorporated into RNA co-transcriptionally by the RNA polymerase [32]. Dinucleoside polyphosphates have been known for more than 50 years and have been detected in all kingdoms of life, including human cells [33]. They are often called alarmones, as their intracellular concentration increases under stress conditions [34]. As Np*_n_*Ns are also present in eukaryotic cells, we hypothesize that they might be incorporated into RNA as non-canonical initiating nucleotides where they can represent an additional layer of information. Moreover, NAD or flavin adenine dinucleotide (FAD) capped RNA was detected in viral particles of the Dengue 2 virus [35], suggesting that non-canonical RNA caps might play a role in the viral life cycle. So far, RNA capped with non-canonical initiating nucleotides, such as NAD, CoA, or Np*_n_*Ns, have not been studied as substrates for any viral encoded enzyme.

Here, we aimed to characterize the SARS-CoV-2 nsp10-nsp16 2′-O-MTase. We prepared a recombinant nsp10-nsp16 complex and analyzed its substrate specificity using LC-MS. First, we tested whether nsp10-nsp16 is capable of methylation of free caps or short hexamer RNA capped with canonical and non-canonical RNA nucleotides. As we did not observe any methylation of the free caps and the methylation of the short hexamer RNA was only partial, we used a longer RNA (35mer). Usually, the methylation of RNA at the 2′-O of ribose is studied by radioactive labelling [24]. We developed a new general technique that can be used for the analysis of any cellular or viral RNA MTase. RNA, which is prepared bearing various caps in vitro, is treated with an MTase and then digested by the Nuclease P1 into nucleotides and caps. The efficiency of the reaction is followed by LC-MS analysis of the digested RNA before and after the methylation reactions. Our analysis showed that nsp10-nsp16 2′-O-MTase can methylate ribose at the 2′ position of RNA capped with m^7^Gp_3_A, Gp_3_A, m^7^Gp_3_G, Gp_3_G, and Gp_4_A. We discovered that the m^7^Gp_3_A-RNA was the best substrate for nsp10-nsp16 in accordance with studies on MTases from other coronaviruses [24,36,37]. We also show that this method is suitable for characterization of MTases inhibitors. As a model compound, we used the pan-MTase inhibitor sinefungin [38] and obtained an IC_50_ value of 138 ± 30 nM.

## 2. Material and Methods

### 2.1. General

All chemicals were either purchased from Merck or Jena Biosciences and used without further purification. Oligonucleotides were purchased from Generi Biotech. m^7^GpppA was synthesized in house according to Baranowski et al. [39] as detailed in the Supplementary Methods.

### 2.2. Protein Expression and Purification

The plasmid encoding for nsp10 and nsp16 proteins was described previously, as was the purification protocol [13]. Briefly, the expression vector was transformed into *E. coli* BL21 cells, and the cells were grown at 37 °C in LB media supplemented with 25 µM ZnSO_4_ until the OD_600_ nm reached 0.5. Subsequently, the expression was induced by IPTG (final concentration: 300 µM), and the temperature was lowered to 18 °C overnight. Cells were harvested, resuspended, and lysed by sonication in lysis buffer (50 mM Tris, pH 8, 300 mM NaCl, 5 mM MgSO_4_, 20 mM imidazole, 10% glycerol, 3 mM β-mercaptoethanol). Proteins were purified by affinity chromatography using the NiNTA agarose (Machery-Nagel), dialyzed against lysis buffer, and digested with Ulp1 protease at 4 °C overnight. The last purification step was size exclusion chromatography at the HiLoad 16/600 Superdex 200 gel filtration column (GE Healthcare) in the SEC buffer (10 mM Tris pH 7.4, 150 mM NaCl, 5% glycerol, 1 mM TCEP). Purified proteins were concentrated to 7 mg/mL and stored in −80 °C until needed.

### 2.3. Preparation of Hexamer

In vitro transcription was performed in a 50 μL mixture containing 80 ng/μL of template DNA (6A), 1 mM NTPs (only those necessary for the RNA production), 1.6 mM Np*_n_*Ns, 5% DMSO, 0.12% triton X-100, 12 mM DTT, 4.8 mM MgCl_2_, and 1x reaction buffer for T7 RNAP and 62.5 units of T7 RNAP (New England BioLabs, NEB). The mixture was incubated for 2 h at 37 °C. After incubation, the samples were injected, without any further purification, in the HPLC, and only the hexamer RNA was collected. The purified RNA was dried on a Speedvac system three times to remove excess triethylammonium acetate (TEAA).

### 2.4. In Vitro Transcription with T7 RNAP for 35mer

In vitro transcription was performed in a 50 μL or 75 μL mixture containing 80 ng/μL of template DNA (35A or 35G) (Table 1), 1 mM NTPs, 1.6 mM Np*_n_*Ns (or ATP or GTP for the control experiments), 5% DMSO, 0.12% triton X-100, 12 mM DTT, 4.8 mM MgCl_2_, and 1x reaction buffer for T7 RNAP and 62.5 units of T7 RNAP (New England BioLabs, NEB). The mixture was incubated for 2 h at 37 °C.

### 2.5. DNAse I Treatment

After the transcription, the DNA template was digested by DNAse I to obtain pure RNA. The transcription mixture (50 μL), 6 μL of 10× the reaction buffer for DNAse I (10 mM Tris-HCl, 2.5 mM MgCl_2_, 0.5 mM CaCl_2_, pH 7.6 at 25 °C, supplied with the enzyme), and 4 units of DNAse I (NEB) were incubated at 37 °C for 60 min. The enzyme was thermally deactivated at 75 °C for 10 min followed by immediate cooling on ice. All samples were purified with RNA Clean and Concentrator^TM^ from ZYMO research and eluted in 25 μL of water for further use.

### 2.6. 5′-Polyphosphatase Treatment

The mixture of capped and uncapped RNA was treated with 20 units of 5′-polyphosphatase (Epicenter) in the solution of 1× buffer in a total volume of 30 μL for 1 h at 37 °C. All samples were purified with RNA Clean and Concentrator^TM^ and eluted in 25 μL of water for Terminator™ treatment.

### 2.7. Terminator™ 5′-Phosphate-Dependent Exonuclease Treatment

The RNA was treated with 1 unit of Terminator ™ 5′-phosphate-dependent exonuclease (Epicenter) in the solution of 1× buffer A in a total volume of 30 μL, and the mixture was incubated at 30 °C for 1 h. All samples were purified with RNA Clean and Concentrator^TM^ and eluted in 15 μL of water for further use.

### 2.8. Nsp10-Nsp16 Reaction for Screening of the Substrates

To test the methyltransferase activity, the cap or the capped RNA samples were divided into two parts. The positive control contained ~3.5 µM (~40 µM for mixture of capped RNA and ppp-RNA) of the RNA, 80 µM (1 mM for mixture of capped RNA and ppp-RNA) of SAM, and 1.5 μM of nsp10-nsp16 in the reaction buffer (40 mM Tris-HCl, 1 mM MgCl2, 5 mM DTT, pH 8 at 25 °C). Nsp10-nsp16 was replaced by water for the negative control. The reactions were performed in 50 µL volume. The mixture was incubated at 30 °C for 1 h (2 h for mixture of capped RNA and ppp-RNA). The enzyme was heat-deactivated at 75 °C for 10 min followed by immediate cooling on ice. The reaction with free caps was analyzed without further purification by HPLC, and capped RNA was digested before analysis by LC-MS.

### 2.9. HPLC Data Collection and Analysis

HPLC was performed using a Waters Acquity HPLC e2695 instrument with PDA detector and with a Kinetex ^®^ XB-C18 column (2.6 μm, 2.1 mm × 50 mm). Mobile phase A was 100 mM TEAA pH 7, and mobile phase B was 100% acetonitrile. The flowrate was kept at 1 mL/min, and the mobile phase composition gradient was as follows: linear decrease from 0% to 12% B (6.5% for dimer analysis) over 20 min, linear decrease to 100% B over 7 min, maintaining 100% B for 3 min, returning linearly to 0% B over 10 min. Waters Fraction Collector III was used for collection of the hexamer RNA.

### 2.10. RNA Digestion for LC-MS

The capped RNA after nsp10-nsp16 reaction was digested using 3 U of Nuclease P1 (Merck) in 50 mM ammonium acetate buffer (pH 4.5) at 37 °C for 1 h. The digested RNA was purified using Amicon-Millipore filters 10 kDa (Merck) to eliminate the Nuclease P1. The flowthrough was dried on a Speedvac system and dissolved in 10 μL of a mixture of acetonitrile (10%) and ammonium acetate (10 mM, pH 9).

### 2.11. LC-MS Data Collection and Analysis

LC-MS was performed using a Waters Acquity UPLC SYNAPT G2 instrument with an Acquity UPLC BEH Amide column (1.7 μm, 2.1 mm × 150 mm, Waters). The mobile phase A consisted of 10 mM ammonium acetate, pH 9, and the mobile phase B of 100% acetonitrile. The flowrate was kept at 0.25 mL/min, and the mobile phase composition gradient was as follows: 80% B for 2 min, linear decrease to 50% B over 4 min (Method Y) or 14 min (Method Z), linear decrease to 5% B over 1 min, maintaining 5% B for 2 min, returning linearly to 80% B over 2 min. For the analysis, electrospray ionization (ESI) was used with a capillary voltage of 1.80 kV, a sampling cone voltage of 20.0 V, and an extraction cone voltage of 4.0 V. The source temperature was 120 °C, the desolvation temperature was 550 °C, the cone gas flowrate was 50 L/h, and the desolvation gas flowrate 250 L/h. The detector was operated in negative ion mode. In total, 8 μL of the dissolved material was injected and analyzed.

### 2.12. Calculation of Methylation Efficiency

MassLynx software was used for the data analysis and the quantification of the relative abundance of all caps. The Area Under the Curve (AUC) for all caps in the positive and negative samples was calculated and normalized for the area of GMP of each negative. The decreasing AUC of the starting material (unmethylated cap) in the nsp10-nsp16-treated sample was compared with the AUC of the starting material (unmethylated cap) in the untreated sample and expressed as a percentage.

### 2.13. Nsp10-Nsp16 Reaction for the Testing of the Inhibitor

For each reaction, ~0.7 µM of pure m^7^Gp_3_A-RNA, 3.6 μM of SAM, 500 nM of nsp10-nsp16, and 5 nM–3 μM of Sinefungine were added to the reaction buffer (40 mM Tris-HCl, 1 mM MgCl2, 5 mM DTT, pH 8 at 25 °C). The mixtures were incubated at 30 °C for 1 h. The enzyme was heat-deactivated at 75 °C for 10 min followed by immediate cooling on ice. The m^7^Gp_3_A-RNA was digested by Nuclease P1 and analyzed by LC-MS.

### 2.14. LC-MS Conditions for the Screening of the Nsp10-Nsp16 Inhibitor

The LC-MS conditions were optimized for the highest signal to noise ratio of the m^7^Gp_3_Am RNA cap. LC-MS was performed using a Waters Acquity UPLC SYNAPT G2 instrument with an Acquity UPLC BEH Amide column (1.7 μm, 2.1 mm × 150 mm, Waters). The mobile phase A consisted of 10 mM ammonium acetate, pH 9, and the mobile phase B of 100% acetonitrile. The flowrate was kept at 0.25 mL/min, and the mobile phase composition gradient was as follows: 80% B for 2 min, linear decrease to 50% B over 4 min, linear decrease to 5% B over 1 min, maintaining 5% B for 2 min, returning linearly to 80% B over 2 min. For the analysis, electrospray ionization (ESI) was used with a capillary voltage of 2.7 kV, a sampling cone voltage of 30.0 V, and an extraction cone voltage of 3.0 V. The source temperature was 120 °C, the desolvation temperature was 500 °C, the cone gas flowrate was 70 L/h, and the desolvation gas flowrate 600 L/h. The detector was operated in positive ion mode. In total, 8 μL of the dissolved material was injected and analyzed.

## 3. Results and Discussion

### 3.1. Methyltransferase Complex of Nsp10-Nsp16 Does Not Methylate Free RNA Caps

In the light of our recent discovery of a new class RNA caps based on dinucleoside polyphosphates (Np*_n_*Ns) [31], we tested whether nsp16-nsp10 may methylate 2′-O position of ribose from various Np*_n_*Ns. We let m^7^Gp_3_A, Gp_3_A, Ap_3_A, m^7^Gp_3_G, Gp_3_G, and Np_4_N (N = A, G) react with nsp10-nsp16 complex in the presence of SAM for 2 h at 30 °C or 37 °C. The reaction mixture was analyzed by HPLC. We did not observe any 2′-O-methylated products. This finding was in an agreement with previously observed SARS-CoV nsp10-nsp16 activity [24] (Figure S1).

### 3.2. Methyltransferase Complex of Nsp10-Nsp16 Partially Methylates the Short m^7^Gp_3_A-RNA

We also tested whether a short RNA (6mer) capped with various dinucleoside polyphosphates can be methylated by this complex. The hexameric RNA was prepared by in vitro transcription with T7 RNA polymerase and free caps. As in vitro transcription with T7 RNA polymerase allows only the preparation of a bigger amount of RNA with sequences typical for T7 bacteriophage, we could not prepare the 5′-terminal sequence (AUUA--) of the SARS-CoV-2 genome. Therefore, we used the template with the T7 promotor φ 2.5, leading to RNA with a sequence of AGGGAA as the model RNA. After HPLC purification, RNA was treated by nsp10-nsp16 complex with SAM for 2 h at 30 °C. The samples were then digested by the nuclease P1 to release 5′-mononucleotides and intact RNA caps and analyzed by HPLC. From all the tested substrates (m^7^Gp_3_A-, Gp_3_A-, NAD-RNA), only m^7^Gp_3_A-RNA was methylated in approximately 20% yield (Figure S2). This experiment shows that the activity of the complex can be observed once a hexameric RNA is used. However, for the development of an inhibitor screening assay, another approach with higher enzymatic activity is desired.

### 3.3. LC-MS Method for the Methyltransferase Activity of Nsp10-Nsp16

Since the hexamer RNA was not an ideal substrate for nsp10-nsp16, we prepared a 35mer RNA with m^7^Gp_3_A cap by in vitro transcription and treated it with the nsp10-nsp16 complex and SAM at 30 °C for 30 min, 1 h, and 2 h. After the indicated times, the samples were digested by Nuclease P1 and analyzed by LC-MS [31]. We followed the disappearance of the unreacted cap (m^7^Gp_3_A) and observed the formation of 2′-O-methylated m^7^Gp_3_A (m^7^Gp_3_Am). The conversion of the reaction was calculated from the difference of the unreacted cap before and after the reaction and confirmed by the formation of the methylated cap. After 2 h, all of the m^7^Gp_3_A cap was converted to m^7^Gp_3_Am (Figure S3A). As the in vitro transcription leads to a mixture of two products—the capped RNA and the uncapped triphosphate RNA (ppp-RNA)—we tested whether the presence of the ppp-RNA, which is not a substrate of the nsp10-nsp16 MTase, can influence the reaction efficiency (Figure S3B). For these purposes, we treated the RNA after in vitro transcription by a 5′ polyphosphatase (cleaves ppp-RNA in form of p-RNA) and terminator exonucleases (degrades p-RNA). This additional treatment leads to pure capped RNA. In the mixture with ppp-RNA, Gp_3_A-RNA was methylated from 37% after 1 h and 59% methylated after 2 h. The yield of the methylation of pure Gp_3_A-RNA with nsp10-nsp16 was somehow higher, at 64% after 1 h and 69% after 2 h. Based on these experiments, we chose the following conditions for the screening of other capped RNAs.: 1 h reaction time and purification of capped RNA from ppp-RNA. We also determined the limit of detection and quantification of our method. The the limit of detection of the methylated product was around 1 µg and the limit of quantification was 2.5 µg if the RNA was not purified before the Nuclease P1 digestion. The purification of RNA led to a decrease of both limits lower than 0.5 µg (Figure S4).

**Figure 2:**
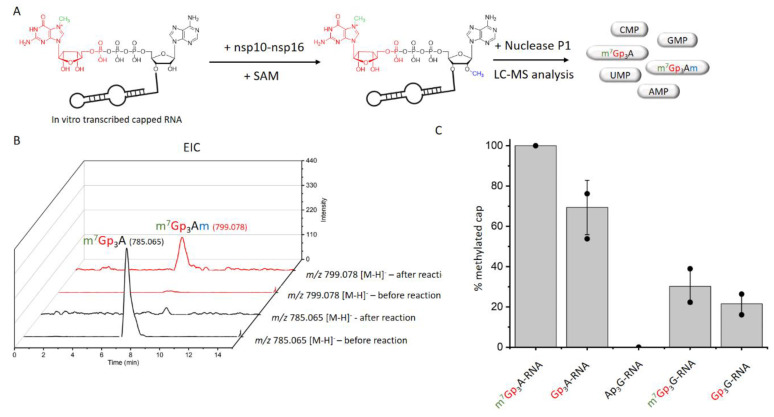
Screening of nsp10-nsp16 activity on non-canonical capped RNA. (**A**) The scheme of experimental set-up. RNA transcribed in vitro was treated by nsp10-nsp16 and SAM, then treated by nuclease P1 and analyzed by LC-MS. (**B**) Extracted Ion Chromatogram (EIC) for *m*/*z* 785.065 and *m*/*z* 799.078 before and after the reaction with nsp10-nsp16, analyzed with method Z. (**C**) The comparison of nsp10 −nsp16 methylation efficiency of various capped RNAs (after the degradation of ppp −RNA).

**Figure 3:**
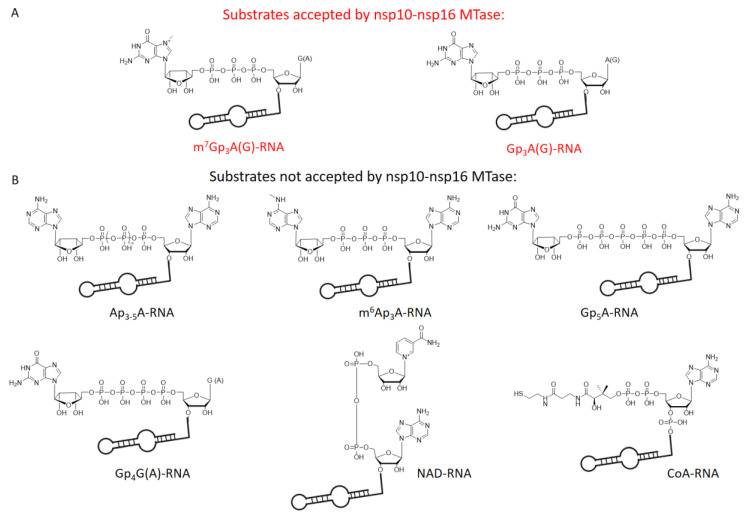
Chemical structures of tested capped RNAs as substrates of nsp10-nsp16 MTase. (**A**) Substrates accepted by nsp10-nsp16 MTase: m^7^Gp_3_A(G)- and Gp_3_A(G)-RNA. (**B**) Substrates not accepted by nsp10-nsp16 MTase: Ap_3-5_A-, m^6^Ap_3_A-, Gp_5_A-, Gp_4_G(A)-, NAD-, and CoA-RNA.

In total, we tested 13 differently capped RNAs (m^7^Gp_3_A, m^6^Ap_3_A, m^7^Gp_3_G, Ap_3-5_N, Gp_3-4_G, NAD, CoA) as substrates for the SARS-CoV-2 nsp10-nsp16 MTase complex. The RNA was prepared as a 35mer by in vitro transcription and treated by 5′ polyphosphatase and terminator exonuclease to degrade the uncapped RNA. Afterward, pure capped RNA was treated by the nsp10-nsp16 complex in the presence of SAM at 30 °C for 1 h. Subsequently, the samples were digested by nuclease P1, and the disappearance of the unreacted cap and formation of the methylated cap were observed (Table S1, Figure 2A). The efficiency of the enzyme activity was calculated by the disappearance of the unreacted cap (Figure 2B). The values were normalized using the guanosine monophosphate (GMP) area under the curve (AUC). Under the conditions optimized for m^7^Gp_3_A-RNA (saturation conditions, when all the substrate is methylated), three other capped RNAs (Gp_3_A-, Gp_3_G-, and m^7^Gp_3_G-RNA) were methylated at the 2′-O position of the +1 nucleotide. All of them were approximately methylated from 70% to 20% (Figure 2C and Figure S5–S9) in comparison with m^7^Gp_3_A-RNA. The yield of the reaction with m^7^Gp_3_G-RNA (30%) may have been negatively influenced by the presence of the side product Gp_3_m^7^G-RNA, which was created by a reverse incorporation of m^7^Gp_3_G during the in vitro transcription. This side product could not be removed from m^7^Gp_3_G-RNA by enzymatic treatment or chromatographic separation. As we encountered the loss of materials during the purification of the capped RNA from the ppp-RNA, the amount of Gp_4_A-RNA may have decreased under the limit of detection. When Ap_3_G was incorporated into RNA in the opposite manner [32], i.e., A was flanking, such capped RNA was not accepted as a substrate of the nsp10-nsp16 MTase at all. Besides Np*_n_*Ns-RNA, which has not been detected in eukaryotic cells so far, we also tested the recently discovered eukaryotic NAD- [26] and CoA-RNA [28] as substrates for the nsp10-nsp16 MTase. Even though the NAD cap has a positive charge similar to that of the canonical m^7^Gp_3_A cap, we did not observe any methylated products. Ap_3-5_A-, m^6^Ap_3_A-, Gp_5_A-, Gp_4_G(A)-, m^7^Gp_4_G-, and CoA-RNA were also not accepted as substrates. Nevertheless, when the methylation reaction of nsp10-nsp16 was performed with a crude transcription mixture (in the presence of ppp-RNA), we also observed the partial methylation of Gp_4_A-RNA (Figure S10). In general, the common pattern shared by all methylated substrates is a polyphosphate bridge with three (to four) phosphates and a flanking G (Figure 3). Moreover, the methylation at the N7 position of the G led to a higher yield of 2′-O methylation of the +1 nucleotide, as both m^7^Gp_3_A-RNA and m^7^Gp_3_G-RNA were better substrates for the nsp10-nsp16 MTase than their non-methylated counterparts Gp_3_A-RNA and Gp_3_G-RNA (Figure 2C). This finding is in good agreement with observations on other coronaviruses, showing that the methylation at the position N7 of the flanking guanosine occurs first and the 2′-O methylation at position +1 follows as the second step.

### 3.4. Non-Radioactive LC-MS Method for Testing of Nsp10-Nsp16 Inhibitors

**Figure 4:**
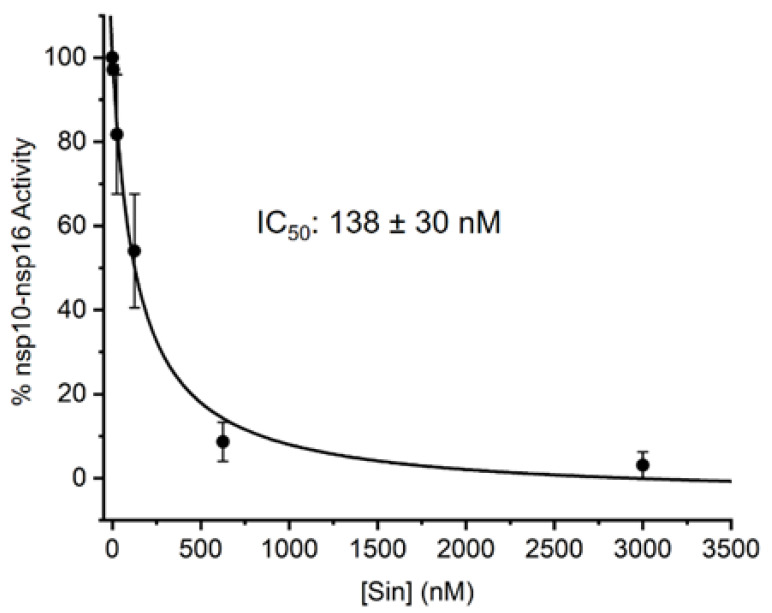
Inhibition curve of Sinefungin. Capped m^7^Gp_3_A-RNA was treated with nsp10-nsp16 and SAM at various concentrations of Sinefungin. After reaction, RNA was cleaved by nuclease P1 and analyzed, and the dimethylated cap (m^7^Gp_3_Am) was quantified by LC-MS. The measurement was performed in triplicate.

So far, the methods used for the screening of inhibitors of RNA MTases were based on radioactive labelling. Here, we took an alternative approach, and we developed an LC-MS based method for assessing the IC_50_ values of the nsp10-nsp16 MTase inhibitors. Our method is general and can be applied to any RNA MTase and RNA of any sequence. We prepared the m^7^Gp_3_A-RNA substrate in vitro and treated it with the nsp10-nsp16 MTase in the presence of SAM and various concentrations of the inhibitor. As a model inhibitor, we chose the pan-MTase inhibitor Sinefungin [40]. We optimized the MTase reaction conditions to reach half conversion of the starting capped RNA. The LC-MS was performed in a positive mode to ensure higher sensitivity of the measurement. Using this method, we were able determined that the IC_50_ value of Sinefungin was 138 ± 30 nM (Figure 4).

## 4. Discussion

Here, we report the development of an LC-MS-based method for the analysis of RNA methylation. Our method is non-radioactive, which is the current trend for safety reasons and is also advantageous for high-throughput screening [41,42]. We applied our method to the nsp16 MTase from SARS-CoV-2 to characterize this important drug target. In total, we tested 14 differently capped RNAs to characterize the substrate specificity of nsp16. As expected, based on the similarity to SARS-CoV nsp16, the best substrate was m^7^Gp_3_A-RNA [24]. However, we observed that RNAs modified with different caps could also be efficiently methylated, namely Gp_3_A—70%, Gp_3_G—22%, m^7^Gp_3_G—30%, and under certain conditions, Gp_4_A—10%. This is, surprisingly, not in contradiction to results obtained on coronaviral MTases, because previous studies on the SARS-CoV nsp16 used short (5mer) RNAs that can be methylated only when m^7^Gp_3_A capped [24]. We observed similar results using short 6mer RNA (Figure S1). This has important implication for the viral life cycle. Here, we show that RNA that is not yet methylated by the nsp14 N7 MTase can be also a substrate for the 2′-O nsp16 MTase, albeit not a good substrate. Nevertheless, this observation challenges the dogma of the step-by-step methylation process of coronaviral RNA (Figure 1). Interestingly, the observation of four different caps (Gp_3_A, Gp_3_G, m^7^Gp_3_G, and Gp_4_A) which are also accepted as substrates for the nsp16 MTase could also play a regulatory role in the stability of viral RNAs. Besides the ~30 kb genomic RNA (serves as mRNA for nsp1-16 proteins), coronaviruses produce up to 10 subgenomic mRNAs that encode structural and accessory proteins [5]. It is tempting to speculate that the methylation of the subgenomic RNAs could serve a regulatory role and control the expression of the coronaviral structural and accessory proteins. However, that is unlikely because it was reported that each positive sense SARS-CoV-2 RNA starts with the same 5′ leader sequence [43]. However, various caps can be on an identical sequence. For several polymerases, it was shown that, if NpnNs are in the proximity of the RNA polymerase, then the RNA polymerase accepts the NPnNs as non-canonical initiating nucleotides [32]. To date, it remains to be confirmed if that is also the case for the coronaviral RdRp. The capability of nsp16 to methylate non-canonically capped RNA may be used for the enzymatic preparation of such RNA for future studies of the biological properties of Np_n_N-RNAs.

The SARS-CoV-2 nsp16 MTase is an important drug target. Often, drug-like candidate molecules are found using high-throughput screening (HTS) [44] and subsequently optimized using medicinal chemistry. If all steps are robotized and a 96- or 384-well format is used for a cost-effective approach, our LC-MS-method could be optimized for HTS using a robotic pipeline and small analytical high-throughput LC-MS instruments [45,46], providing a new tool for drug discovery against COVID-19.

Recently, an alternative MS method was published for the analysis of methylation of 25 mer canonically capped RNA by nsp16 MTase without Nuclease P1 digestion [14]. This method uses chemically prepared oligoes as the substrate, while we use enzymatically prepared RNA. Taken together, our LC-MS based approach and an in-depth analysis show that SARS-CoV-2 nsp16 has a broader substrate specificity than previously believed. In particular, the ability of nsp16 to use a non-methylated Gp_3_A has important implications for the viral life cycle because it reveals that nsp16 can, in principle, act before the nsp14 N7 MTase.

## Figures and Tables

**Table 1:** Sequences of template DNA used for *in vitro* transcription. T7 promoter sequence is underlined, first transcribed base is in bold.

Name	Sequence
6A	5′-CAGTAATACGACTCACTATT**A**GGGCT-3′
35A	5′-CAGTAATACGACTCACTATT**A**GGGAAGCGGGCATGCGGCCAGCCATAGCCGATCA-3′
35G	5′-CAGTAATACGACTCACTATA**G**GGGAAGCGGGCATGCGGCCAGCCATAGCCGATCA-3′

## Supplementary Materials

The following are available online at https://www.mdpi.com/article/10.3390/v13091722/s1, ten supplementary figures (Figures S1–S10), one supplementary table (Table S1), and three supplementary spectrums.
