# Supplementary material for: Substrate Specificity of SARS-CoV-2 Nsp10-Nsp16 Methyltransferase
Source: Viruses. 2021 Aug 30;13(9):1722. doi: 10.3390/v13091722 (PMC8472550; doi:10.3390/v13091722)
Supplement: Supplementary file 1 [file viruses-13-01722-s001.zip › viruses-1340374-supplementary.pdf]

Supplementary information

Substrate specificity of SARS-CoV-2 nsp10-nsp16 methyltransferase

Roberto Benoni [1,#], Petra Krafcikova[1,#], Marek R. Baranowski[2], Joanna Kowalska[2], Evzen Boura[1*], Hana Cahová[1*]

Synthesis and spectroscopic characterization of m^7GpppA

m^7GpppA (2160 mOD, 0.101 mmol, 72%) was synthesized by coupling between adenosine 5'-diphospahte triethylammonium salt (ADP) and N^7-methylguanosine 5'-phosphorimidazolide sodium salt (m^7GMP-Im), which were both prepared as described earlier (Baranowski, J. Org. Chem. 2015, 80, 3982−3997).

ADP (2100 mOD, 0.140 mmol) was mixed with DMSO (0.8 mL) and $ZnCl_2$ (228 mg, 1.68 mmol) and left for 10 min under vigorous stirring at room temperature. Then, m^7GMP-Im (3985 mOD, 0.350 mmol) was added and the reaction progress was monitored by RP HPLC until total conversion of ADP to m^7GpppA was observed. The reaction was quenched by addition of a solution of Na_2EDTA (8−10 mmol) and $NaHCO_3$ (~35 mmol) in deionized water (10 ml). The product was purified by DEAE Sephadex chromatography using a linear gradient of triethylammonium bicarbonate buffer (0.9 M) in water. Fractions containing the desired products (as verified by UV, HPLC, and MS analysis) were mixed together and evaporated under reduced pressure with repeated additions of 96% and, then, 99.8% ethanol (to decompose TEAB and remove residual water, respectively). The product was additionally purified by semi-preparative RP HPLC on a VisionHT C18 HighLoad column (Dr. Maisch, 250 mm x 20 mm, 10 µm, flow rate 5 mL/min) using a linear gradient of acetonitrile in 0.05 M ammonium acetate buffer (pH 5.9). The final product was lyophilized three times from water and analyzed by NMR and electrospray MS (ESI-).

^{1}H NMR (500 MHz, D_2O): δ 8.40 (s, 1H), 8.14 (s, 1H), 6.00 (d, *J* = 6.0 Hz, 1H), 5.86 (d, *J* = 3.4 Hz, 1H), 4.65 (t, *J* = 6.0 Hz, 1H), 4.51 – 4.47 (m, 2H), 4.42 – 4.24 (m, 8H), 3.99 (s, 3H); ^{31}P NMR (202 MHz, D_2O) δ -10.35 – -10.89 (m, 2P), -22.20 (t, *J* = 19.4 Hz, 1P); ^{31}P NMR {^{1}H} (202 MHz, D_2O) δ -10.63 (d, *J* = 19.3 Hz, 1P), δ -10.67 (d, *J* = 19.3 Hz, 1P), -22.20 (t, *J* = 19.3 Hz, 1P); MS (ESI-)

Table S 1: Table of calculated and detected m/z values in LC-MS analysis of digested RNA before and after reaction with nsp10-nsp16.

cap	calculated mass	detected mass
m^7Gp_3A	785.085	785.078
m^7Gp_3Am	799.101	799.089
m^6Ap_3A	769.09	769.078
m^6Ap_3Am	783.105	n.d.
m^7Gp_3G	400.036	400.032
m^7Gp_3Gm	407.044	407.042
Ap_3A	755.074	755.076
Ap_3Am	769.090	n.d.
Ap_4A	417.016	417.022
Ap_4Am	424.024	n.d.
Ap_5A	457.000	457.000
Ap_5Am	464.007	n.d.
Ap_3G	771.069	771.062
Ap_3Gm	785.085	785.078
Ap_4G	425.014	425.016
Ap_4Gm	432.022	432.028
Ap_5G	464.997	464.995
Ap_5Gm	472.005	n.d.
Gp_3G	787.063	787.060
Gp_3Gm	801.08	801.065
Gp_4G	433.011	433.005
Gp_4Gm	440.019	n.d.
NAD	662.103	662.087
NADm	676.119	n.d.
CoA	686.144	686.119
CoAm	700.16	n.d.

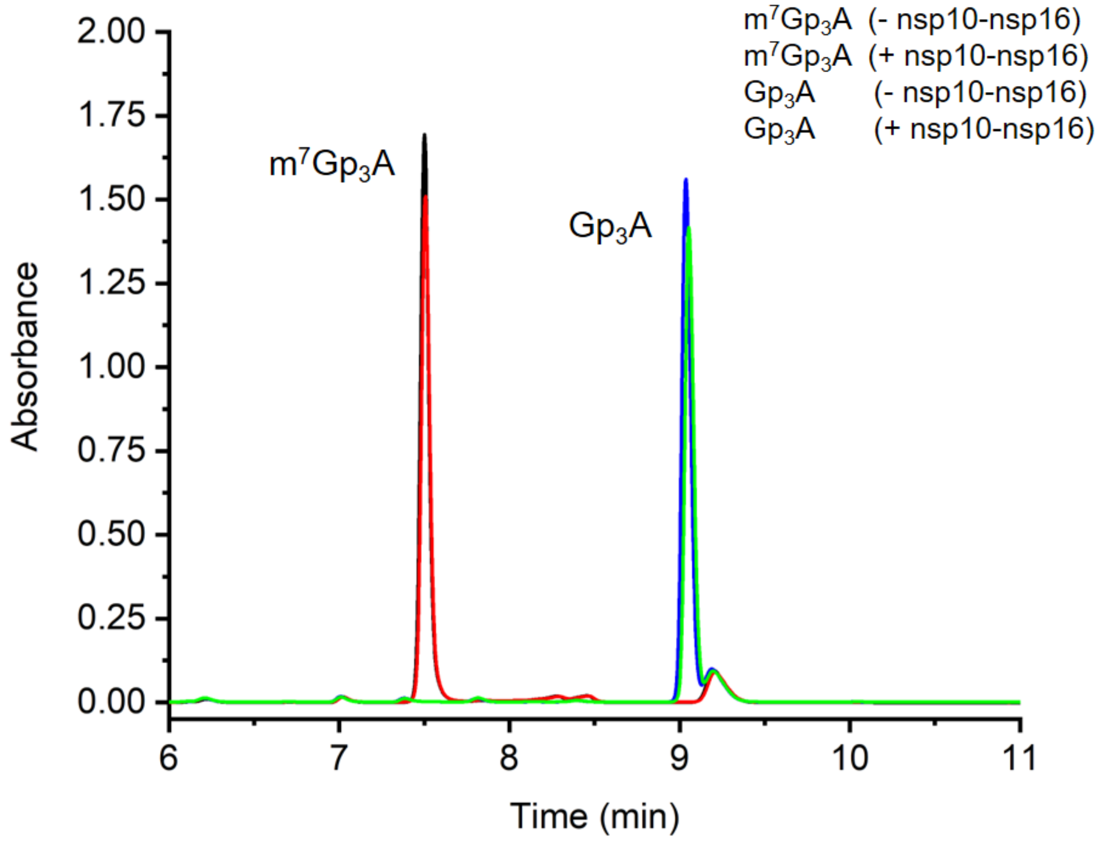

Figure S 1: The HPLC chromatogram of free RNA caps before and after the treatement with nsp10-nsp16.

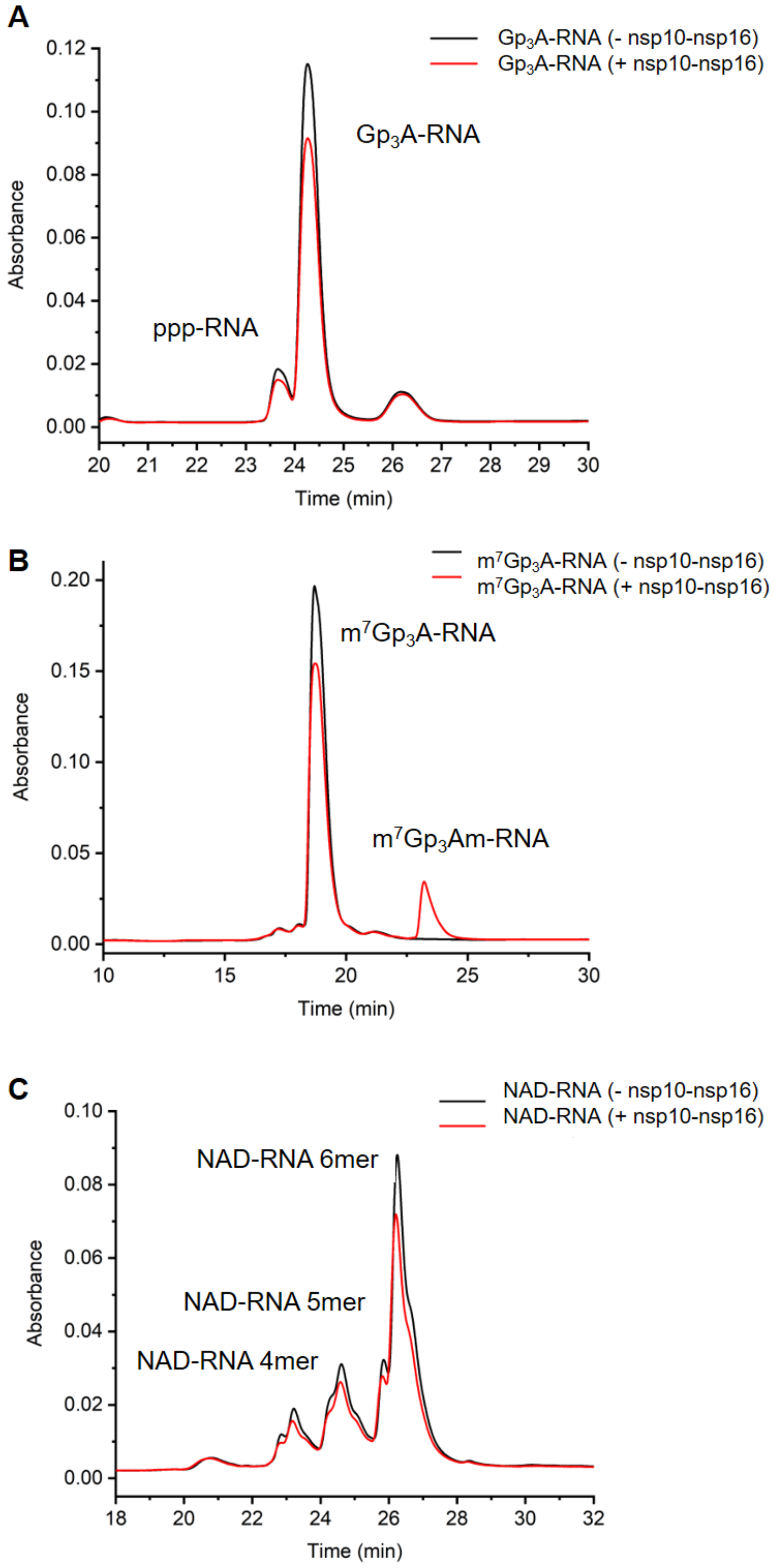

Figure S 2: HPLC chromatograms of hexamer RNA capped with Gp_3A (A), m^7Gp_3A (B) and NAD (C) before and after the treatement with nsp10-nsp16.

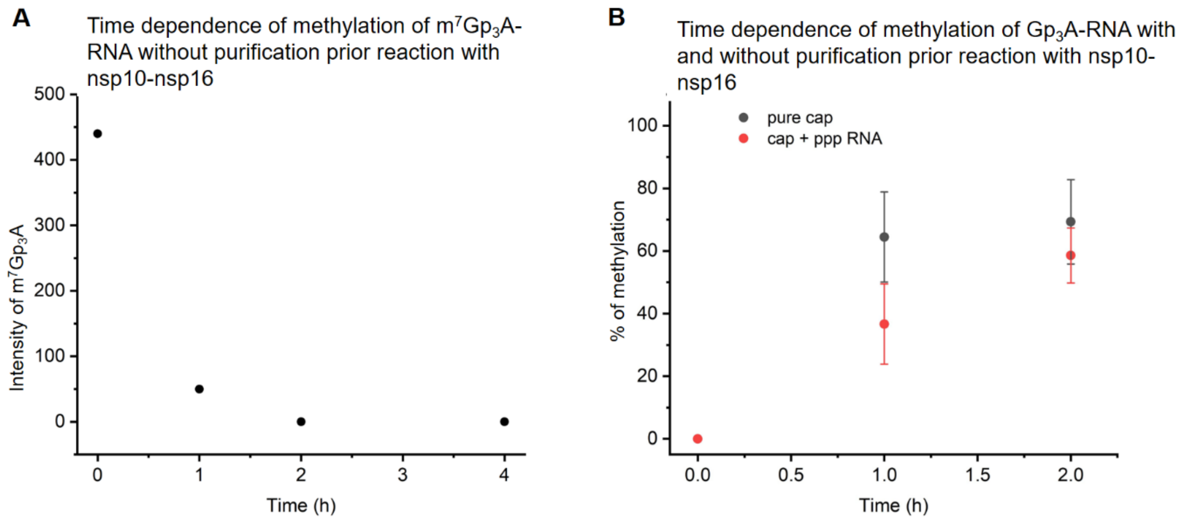

Figure S 3: Time dependence of the methylation of m^7Gp_3A-RNA by nsp10-nsp16 in the presence of SAM without purification of RNA prior reaction with nsp10-nsp16 (A). Comparison of methylation of Gp_3A-RNA with and without purification from ppp-RNA prior reaction with nsp10-nsp16 (B).

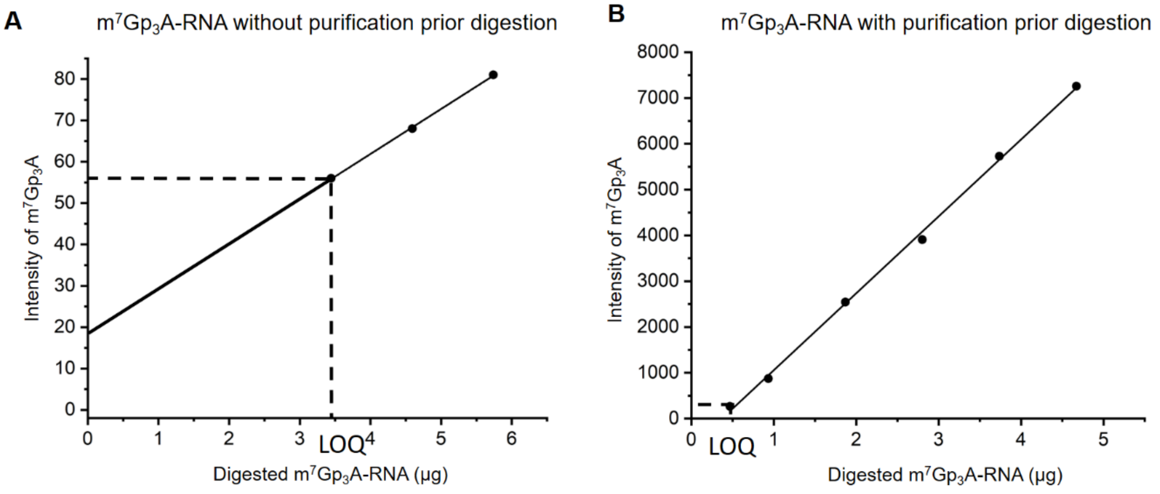

Figure S 4: Determination of the limit of detection and quantification of the method for m^7Gp_3A using m^7Gp_3A-RNA without purification prior to Nuclease P1 digestion (A) or purified RNA using RNA Clean and Concentrator™ kit prior to Nuclease P1 digestion (B).

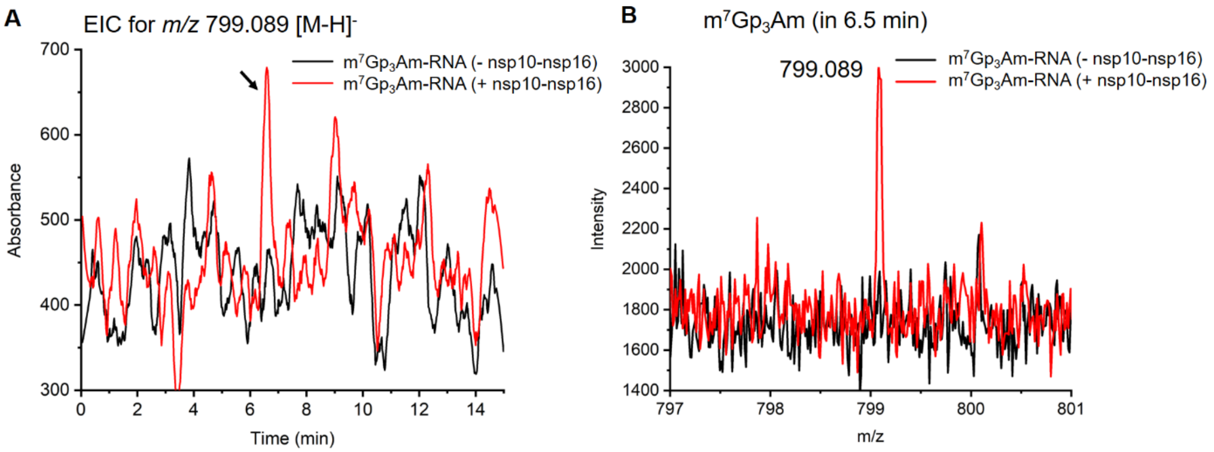

Figure S 5: LC-MS analysis of digested RNA before and after nsp10-nsp16 treatment, using method Y. A) Extracted Ion Chromatogram (EIC) of m/z 799.089 from m⁷Gp₃A-RNA before and after the nsp10-nsp16 treatement. B) MS spectrum of the m/z 799.089 corresponding to m⁷Gp₃Am before and after the nsp10-bsp16 treatment.

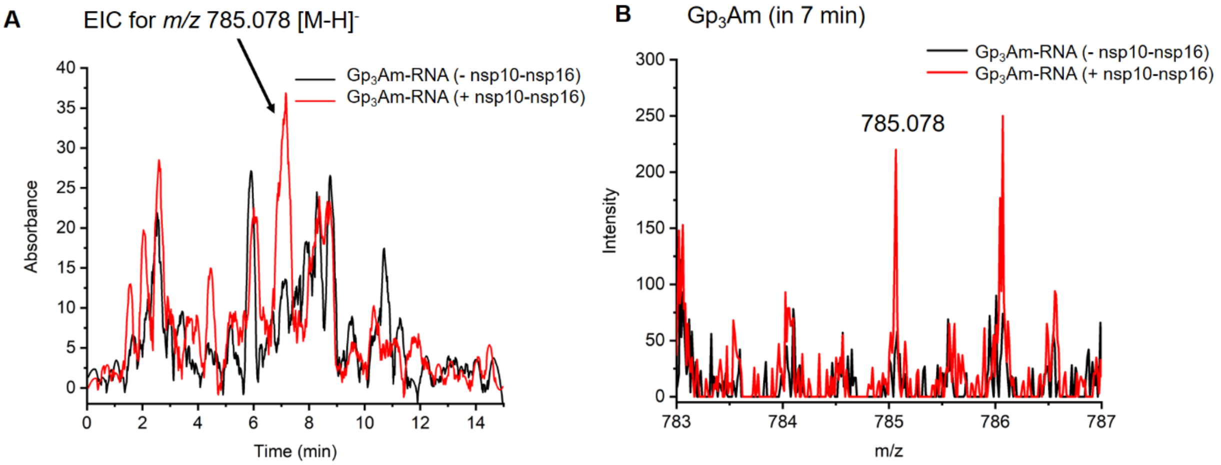

Figure S 6: LC-MS analysis of digested RNA before and after nsp10-nsp16 treatment, using method Y. A) Extracted Ion Chromatogram (EIC) of m/z 785.078 from Gp₃A-RNA before and after the nsp10-nsp16 treatement. B) MS spectrum of the m/z 785.078 corresponding to Gp₃Am before and after the nsp10-bsp16 treatment.

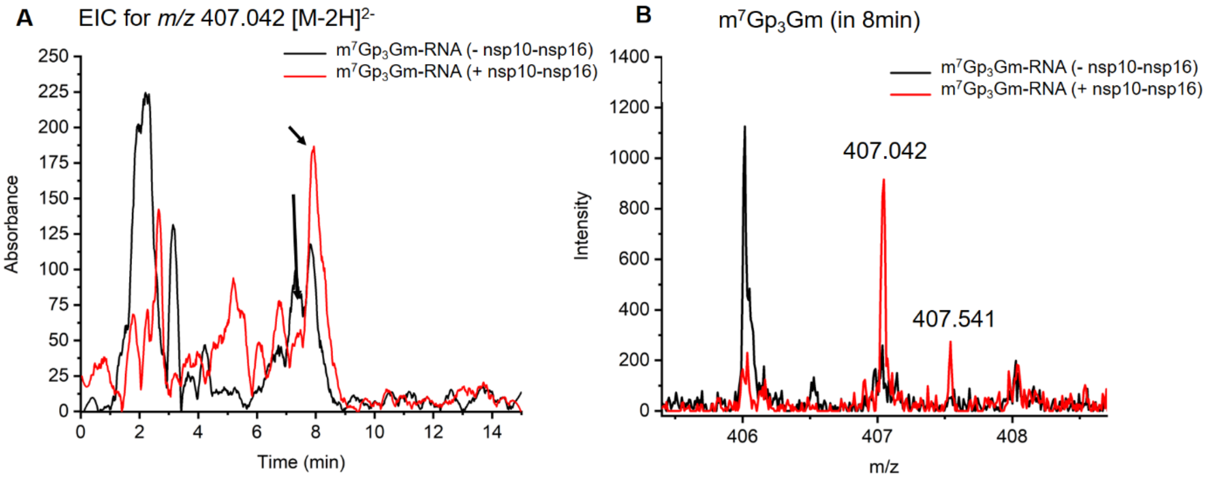

Figure S 7: LC-MS analysis of digested RNA before and after nsp10-nsp16 treatment, using method Y. A) Extracted Ion Chromatogram (EIC) of m/z 407.042 from m^{7}Gp$_3$G-RNA before and after the nsp10-nsp16 treatement. B) MS spectrum of the m/z 407.042 corresponding to m^{7}Gp$_3$Gm before and after the nsp10-bsp16 treatment.

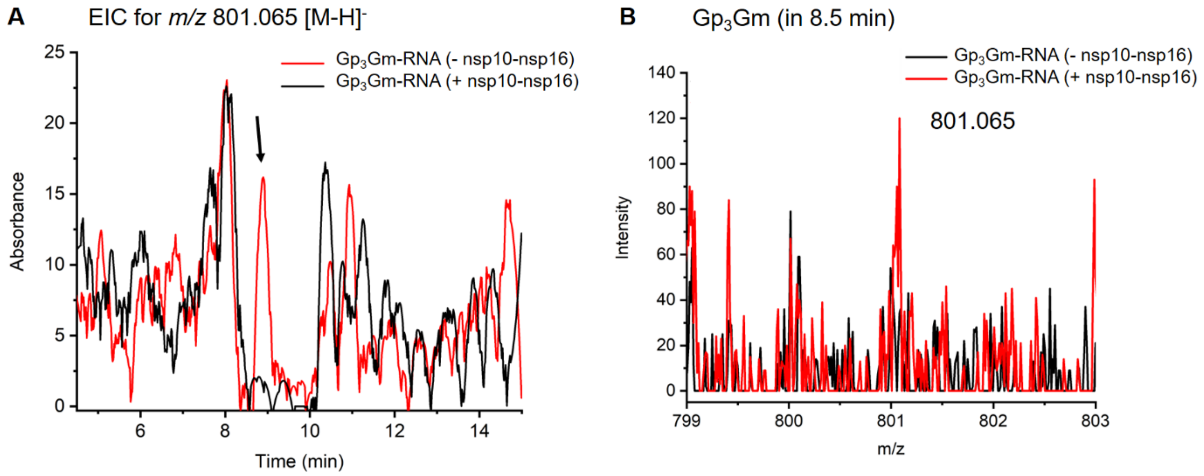

Figure S 8: LC-MS analysis of digested RNA before and after nsp10-nsp16 treatment, using method Y. A) Extracted Ion Chromatogram (EIC) of m/z 801.065 from Gp$_3$G-RNA before and after the nsp10-nsp16 treatement. B) MS spectrum of the m/z 801.065 corresponding to Gp$_3$Gm before and after the nsp10-bsp16 treatment.

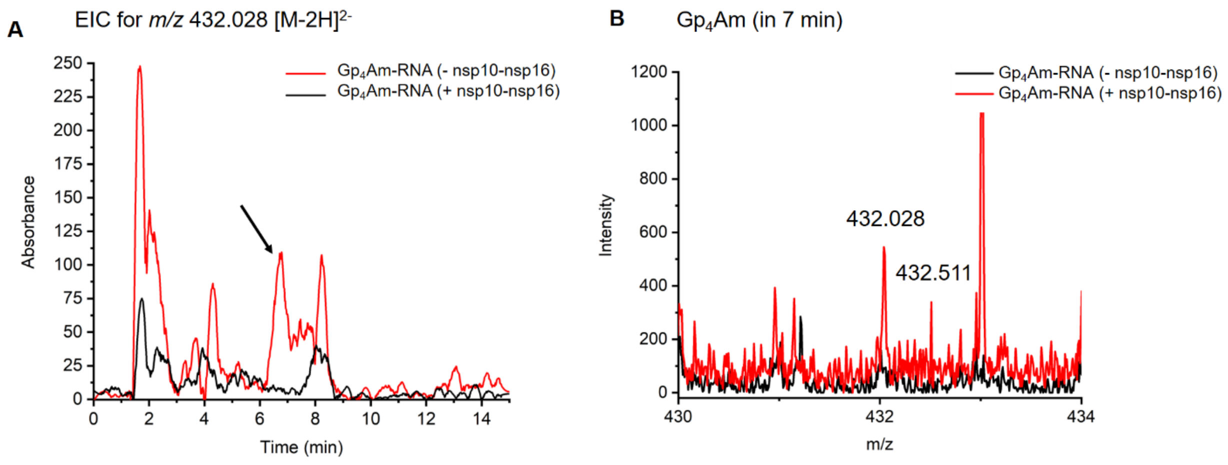

Figure S 9: LC-MS analysis of digested RNA before and after nsp10-nsp16 treatment, using method Y. A) Extracted Ion Chromatogram (EIC) of m/z 432.028 from Gp₄A-RNA before and after the nsp10-nsp16 treatement. B) MS spectrum of the m/z 432.028 corresponding to Gp₄Am before and after the nsp10-bsp16 treatment.

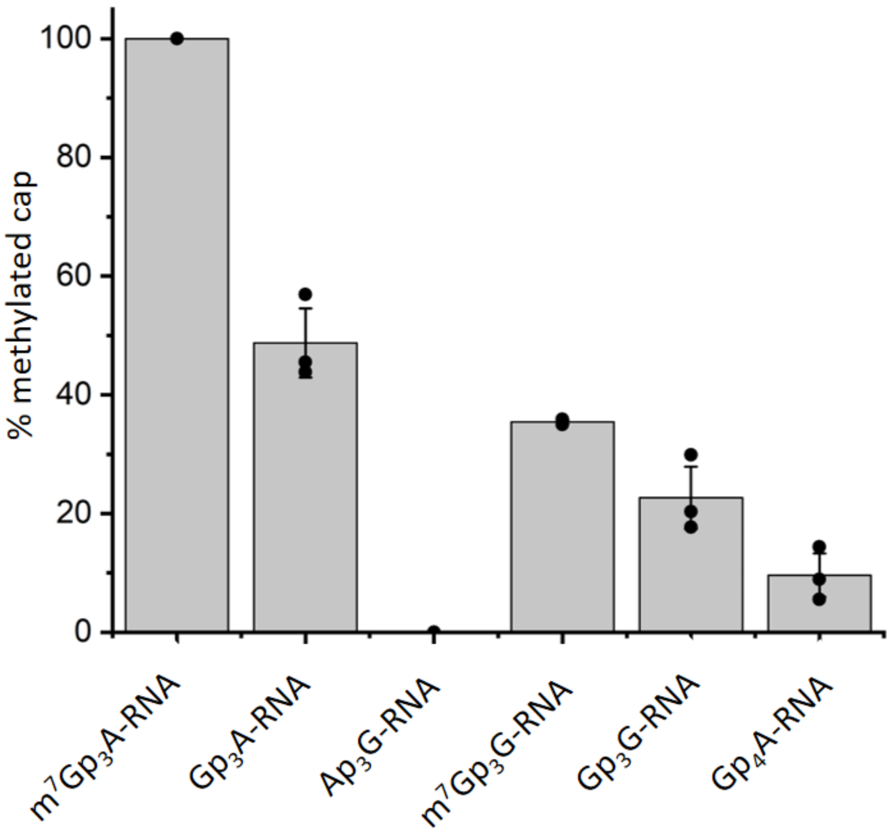

Figure S 10: The comparison of nsp10-nsp16 methylation efficiency of various capped-RNAs without purification from ppp-RNA prior the methylation reaction.

NMR spectra

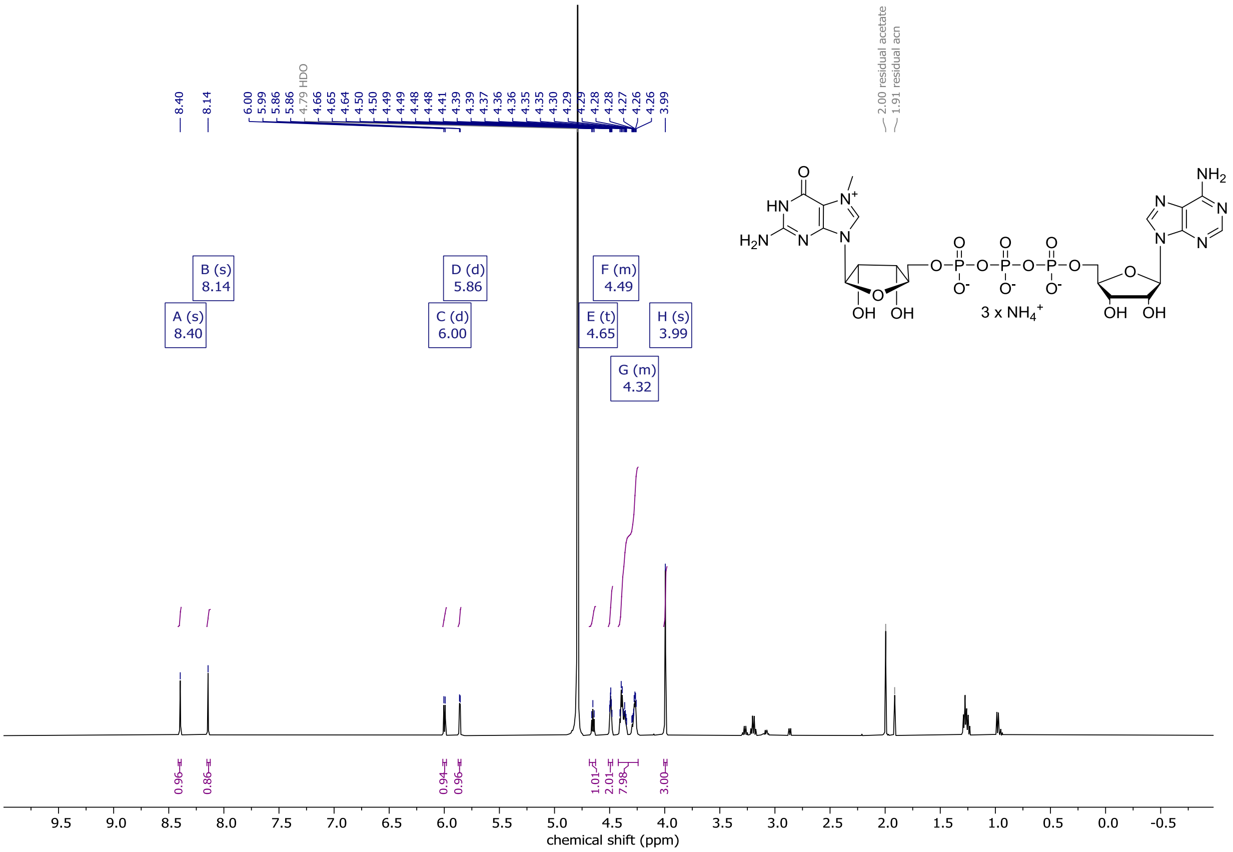

Spectrum 1. ^{1}H NMR of m^{7}GpppA.

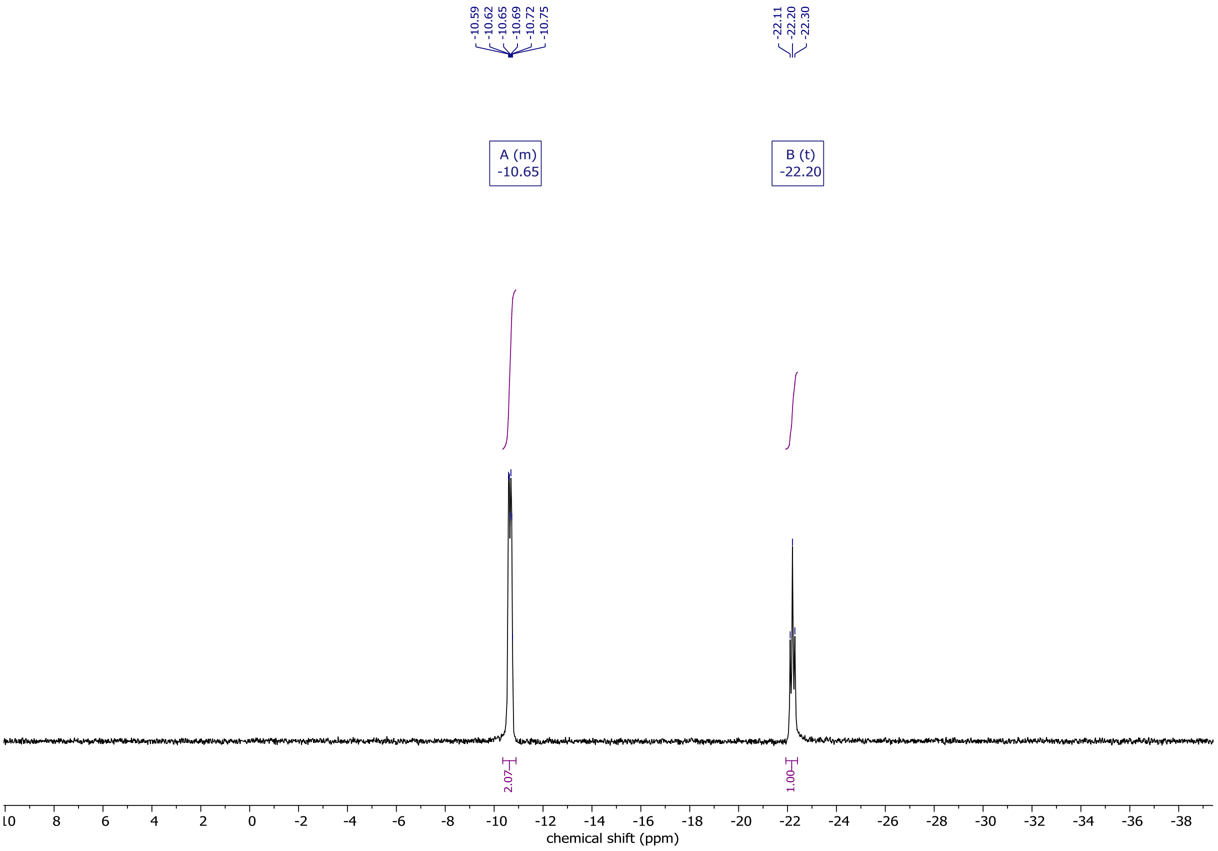

Spectrum 2. ^{31}P NMR of m^7GpppA.

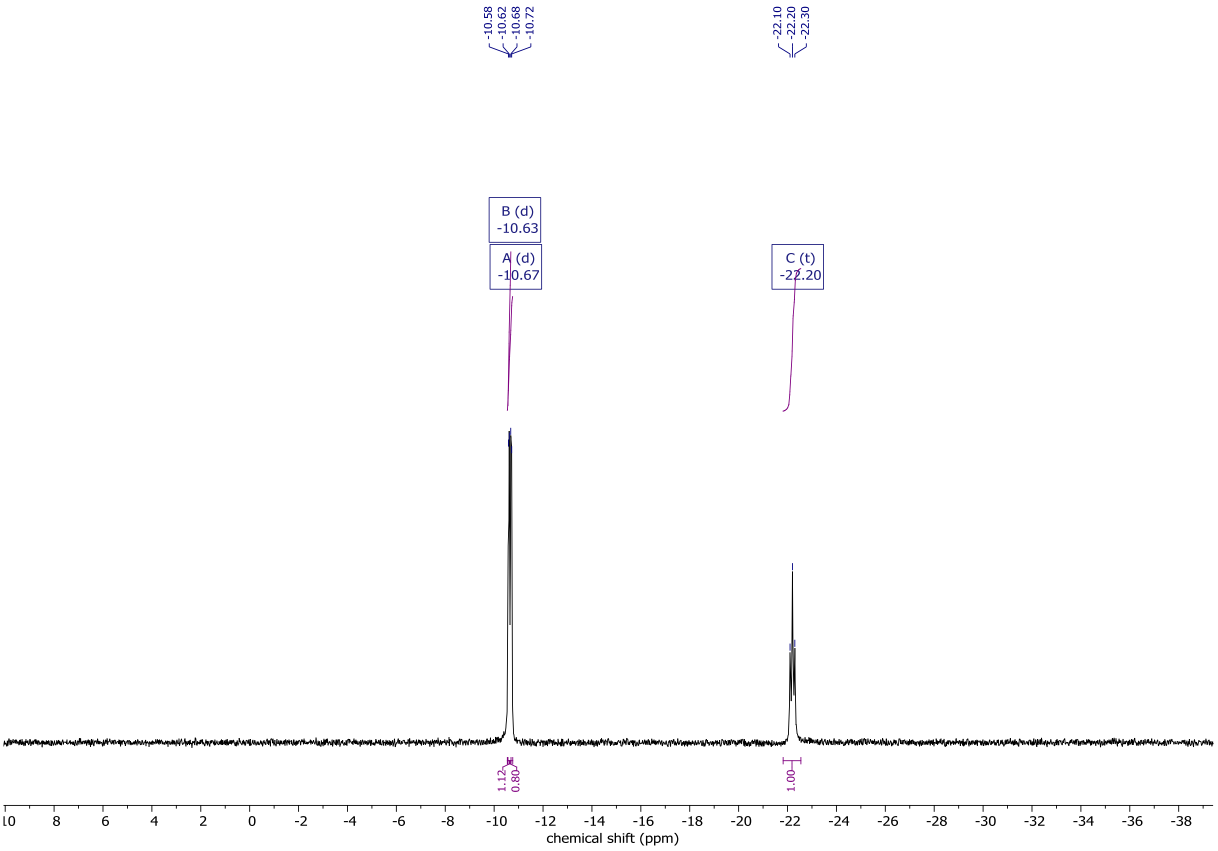

Spectrum 3. $^{31}P\ \{^1H\}$ NMR of m^7GpppA.
